# Reduced State Psychiatric Hospital Capacity and Increased Restraint Use: A Case Series From a Community Psychiatric Hospital

**DOI:** 10.7759/cureus.95147

**Published:** 2025-10-22

**Authors:** Minaal Khan, Prutha Patel, Yash K Desai, Sebhat Erqou, Partam Manalai

**Affiliations:** 1 Psychiatry, Liberty University College of Osteopathic Medicine, Lynchburg, USA; 2 Psychiatry, Mary Washington Hospital, Fredericksburg, USA; 3 Psychiatry, Edward Via College of Osteopathic Medicine, Blacksburg, USA; 4 Cardiology, Mary Washington Healthcare, Fredericksburg, USA; 5 Psychiatry, Mary Washington Healthcare, Fredericksburg, USA

**Keywords:** 30-day readmission, covid-19, deinstitutionalization, healthcare worker safety, physical restraints, schizophrenia spectrum

## Abstract

Severe mental illness (SMI) is a significant and persistent public health challenge in the United States. In recent decades, shifts in mental health policy regarding deinstitutionalization have altered how long-term psychiatric care is delivered, often leaving individuals with some of the gravest forms of SMI without sustained support. This paper presents a case series of three patients, advocating for a limited increase in the number of state-funded hospitals in the Commonwealth of Virginia. These patients were repeatedly admitted for treatment-resistant schizophrenia. Once stabilized in an acute care psychiatric hospital, they would be discharged to home and often stop treatment, leading to readmission. After multiple readmissions, it was concluded by clinicians that they may be better suited for a long-term psychiatric facility in order to achieve sustained remission from schizophrenia. However, several long-term psychiatric hospitals had significantly decreased their capacity. This led to higher acuity in an acute care hospital, as observed by a greater number of restraints being utilized, leading to difficulties in providing adequate care for all at the hospital. This case series underscores the importance of a more personalized approach to healthcare, as opposed to the large, poorly regulated institutions of the past in the United States that often subjected patients to inhumane treatment.

## Introduction

The World Health Organization (WHO) reports that approximately one in eight individuals worldwide experiences some form of mental illness, with 280 million people living with depression, 40 million with bipolar disorder, and 24 million with schizophrenia [1]. In the United States, approximately 57.8 million individuals were diagnosed with a mental illness in 2021, reflecting a wide range of severity [2]. Severe mental illness (SMI) is defined as a mental, behavioral, or emotional disorder that results in significant functional impairment, substantially interfering with or limiting one or more major life activities. In the U.S. alone, 14.1 million individuals over the age of 18 suffer from SMI. This condition is associated with a range of medical, social, and economic consequences that impact not only the individuals affected but also their families and support networks [2,3].

Given the chronic, relapsing nature of SMI, many individuals require acute inpatient care. However, there is a subset of patients whose condition does not improve in such settings, necessitating long-term care. Historically, these patients would have been admitted to state psychiatric facilities. Yet, due to the process of deinstitutionalization, these options have become increasingly scarce, particularly in the Commonwealth of Virginia, where such services continue to decline rapidly. We present three cases where the patients would be better served in a long-term care facility that provides patient-centered care and supports dignified recovery without frequent re-hospitalizations. These individuals were re-admitted (with similar presentations each time) and were well known to the facility. We will reflect on the state of long-term psychiatric inpatient facilities in the Commonwealth of Virginia, followed by a discussion of the adverse outcomes resulting from this shortage.

## Case presentation

Patient 1

This was a male patient in his late twenties, admitted involuntarily for the management of severe psychosis. He met the diagnostic criteria for schizoaffective disorder, bipolar type; posttraumatic stress disorder (PTSD); and over-the-counter antihistamine use disorder. During his initial hospitalization, the patient exhibited significant behavioral dysregulation, including multiple altercations with peers and repeated threats of aggression toward staff and hospital property. He required one-to-one observation and multiple administrations of emergency medications at various doses consistent with accepted clinical standards including: first generation antipsychotics (oral and injectable haloperidol, oral perphenazine); second generation antipsychotics (oral olanzapine, injectable ziprasidone, oral quetiapine); benzodiazepine (oral and injectable lorazepam, diazepam, oral clonazepam, temazepam); antihistamine (oral and injectable diphenhydramine); and anticholinergic medications (oral and injectable benztropine). After pharmacologic interventions over objection were authorized through judicial proceedings, the patient was trialed on several antipsychotic agents, ultimately remaining hospitalized for 40 days. He was readmitted two days after his first discharge, due to the recurrence of symptoms. His second admission was similarly marked by frequent behavioral codes and emergency medication administration (as noted above), and he was discharged after 14 days.

Patient 2

This was a male patient in his sixties who presented with acute mania and was admitted under involuntary status after approximately one year of community stability. He carried a diagnosis of schizoaffective disorder, bipolar type, and was later civilly committed. Although he had previously demonstrated a robust therapeutic response to fluphenazine (firstly stabilized with oral 10 mg fluphenazine followed by fluphenazine decanoate 50 mg monthly), he declined this treatment during the current episode, prompting the administration of antipsychotic medications over objection, as approved by the court. He was initiated on long-acting aripiprazole but subsequently developed seizures, requiring temporary transfer to the medical unit for further evaluation and stabilization. Upon return to the psychiatric unit, he continued to exhibit significant aggression toward both staff and peers, resulting in prolonged room restriction throughout his 51-day hospitalization. He required frequent intramuscular injections of chlorpromazine (escalating dose starting at 25 mg at the peak of 300 mg deltoid injection) for behavioral management. He was readmitted 10 months later for a similar episode, with stabilization achieved over a 57-day hospitalization using a long-acting aripiprazole monohydrate 300 mg injection monthly. He was again hospitalized one month later for another recurrence, with clinical stabilization achieved over 45 days using haloperidol decanoate total monthly dose of 400 mg long-acting injection.

Patient 3

This was a male patient in his early thirties admitted involuntarily for an acute exacerbation of schizoaffective disorder, bipolar type. He was subsequently committed due to the severity of his symptoms. During his 32-day hospitalization, the patient demonstrated sexually inappropriate behavior and aggression, necessitating one-to-one observation and multiple administrations of emergency intramuscular medications with dose ranges consistent with evidence-based practice including: first generation antipsychotics (oral and injectable haloperidol); second generation antipsychotics (oral olanzapine, oral and injectable ziprasidone); benzodiazepine (oral and injectable lorazepam, diazepam, oral clonazepam, temazepam); antihistamine (oral and injectable diphenhydramine); and anticholinergic medications (oral and injectable benztropine). Clinical stabilization was achieved with a regimen including olanzapine 20 mg nightly, fluphenazine decanoate 25 mg long-acting monthly injection, valproic acid 500 mg three times daily, allowing for discharge. However, he was readmitted four days later with a recurrence of symptoms, including aggression toward family members. On this second admission, the patient responded to a haloperidol decanoate 100 mg long-acting injection, along with valproic acid 500 mg twice daily, sertraline 50 mg daily, along with lurasidone 120 mg daily, returning to his premorbid baseline by day 27 of hospitalization.

## Discussion

In our selected case series, Patient 1 would likely benefit from a prolonged stay (perhaps exceeding a year), as he previously responded well to stabilization at a state hospital and transitioned to the community successfully once stabilized. Patient 2 would likely require hospitalization for less than a year. Had he been admitted to a state hospital during his second admission, he may have achieved stability more quickly. Patient 3, however, will likely need episodic admissions to a state hospital for less than a year once his symptoms fail to improve in acute care psychiatric settings.

On June 4, 1770, the Commonwealth of Virginia passed a bill authorizing the establishment of the first public facility dedicated to those living with mental illness [4]. In 1773, near the College of William and Mary, the Public Hospital for Persons of Insane and Disordered Minds (renamed Eastern Lunatic Asylum in 1841 and Eastern State Hospital in 1894) was opened. It took another 52 years for the establishment of a second psychiatric facility, Western State Hospital, in 1825. Under Dr. John Galt at the Eastern Lunatic Asylum (ELA), 125 patients received treatment modalities almost a century ahead of their time. Dr. Galt (who may have eventually died by suicide) introduced Moral Management to ELA, advocating for a vast array of treatment modalities, including the deinstitutionalization of patients once they recovered [4].

Another prominent figure in the history of mental health reform, Dorothea Dix, spearheaded the movement for publicly funded institutions for mental illness and played a key role in establishing more than 30 psychiatric hospitals, including Saint Elizabeth's Hospital in 1852 [5]. As the number of psychiatric hospitals, residents, and the scope of admissions increased, many of these institutions became overcrowded and underfunded, leading to the adoption of extreme and unethical measures. Following the end of World War II, reports of inhumane conditions in these hospitals prompted a reevaluation of mental health care [6]. The National Mental Health Act of 1946 paved the way for the establishment of the National Institute of Mental Health (NIMH) in 1949 [7]. Despite these efforts, however, the treatment modalities and outcomes within these large institutions remained disappointing, while the number of patients continued to grow, culminating in 550,000 patients in 1955 [8].

With the aim of improving the mental health services available to the American public, Congress passed the Community Mental Health Act (CMHA), signed by President Kennedy in 1963 [9]. On October 31, 1963, President Kennedy stated, “This bill will expand our knowledge, provide research facilities to determine the cause of retardation, establish university-related diagnostic treatment clinics, and permit the construction of community centers for the care of the mentally disabled. For the first time, parents and children will have access to comprehensive facilities to diagnose and either cure or treat mental retardation. For the first time, research centers will bring together teams of experts from various fields. For the first time, State and Federal governments and voluntary organizations will be able to coordinate their manpower and facilities in a unified effort to cure and treat this condition” [10]. This legislation marked a significant shift in the approach to mental health care and support services in the United States.

Although the intention of the CMHA was noble, the outcome proved disappointing. President Johnson signed the bill establishing Medicare and Medicaid into law on July 30, 1965 [11], but the “Institutions for Mental Diseases” (IMD) exclusion prevented federal funding for care provided in inpatient psychiatric hospitals [12]. Any hospital with more than 16 psychiatric beds was classified as an IMD, which made it difficult for psychiatric hospitals to receive federal reimbursement. As a result, while the number of psychiatric beds in specialized institutions dwindled, expanding psychiatric care in general hospitals became less appealing to private hospitals. These changes led to many patients in need of long-term care being discharged inappropriately, forcing individuals with SMI into the legal system. Currently, the two largest providers of mental health services in the United States are prisons and jails [11].

**Figure 1:**
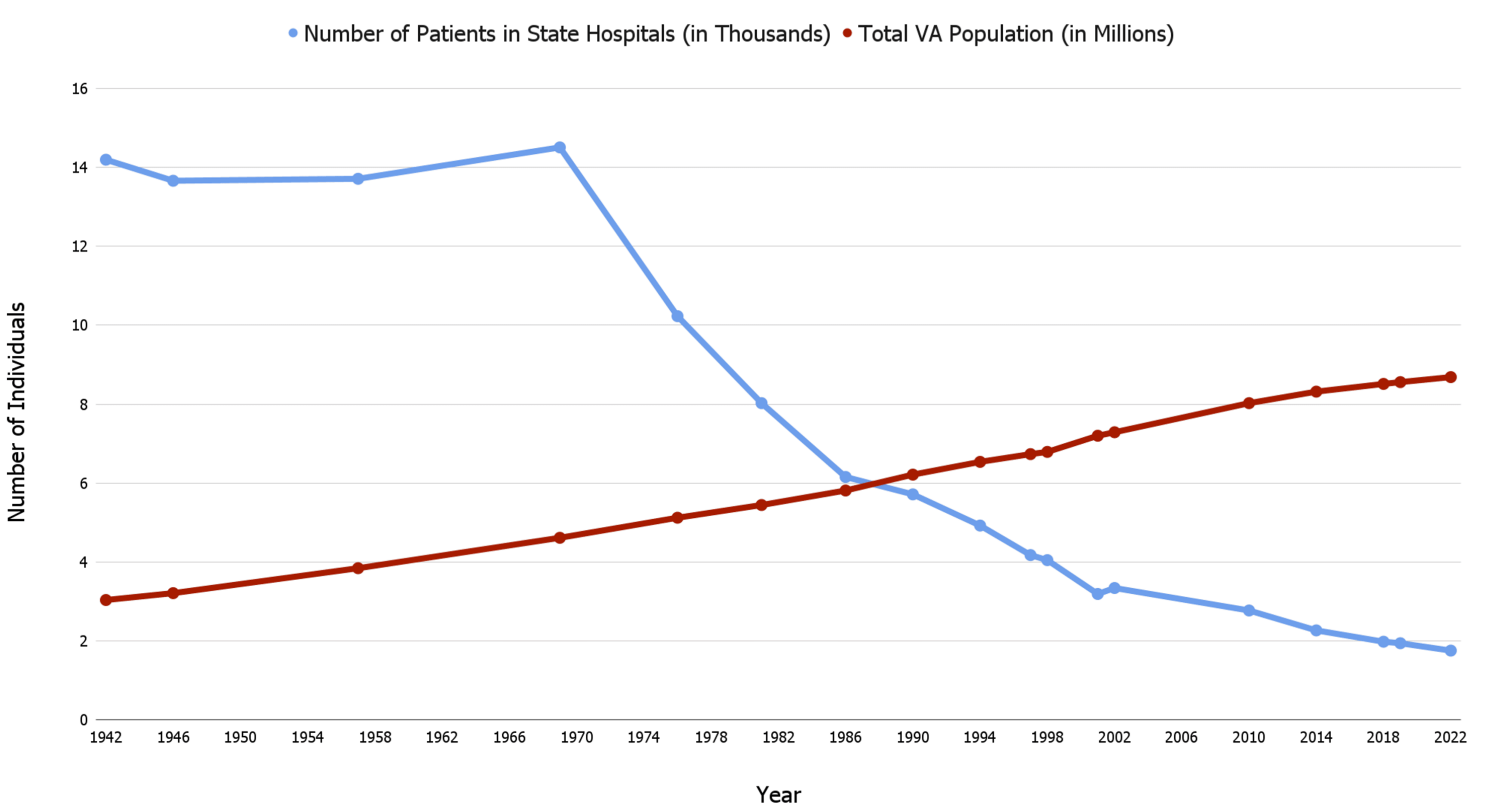
Decline in Virginia's inpatient census as compared to the total population of Virginia as a function of time Blue line: Number of patients in state hospitals (in Thousands); Red line: Total population of Virginia (in Millions) Figure created by authors using Google Sheets (Google Drive, California, US) using data obtained from [13].

Similar to the rest of the United States, in the Commonwealth of Virginia, the total daily census has declined steadily, from 14,501 patients for 3.038 million Virginians to 1,757 patients for 8.675 million Virginians (Figure 1) [13].

To further complicate the plight of limited public psychiatric beds for patients needing long-term care, state hospitals have had to prioritize admitting forensic patients, thereby decreasing opportunities for civil patients [14]. With the onset of the SARS COVID-19 pandemic, the opportunity to admit civil patients to psychiatric hospitals has become nearly impossible. As a result, patients often remain in acute care psychiatric hospitals for extended periods while awaiting admission to state facilities. Under pressure, state facilities expedite the discharge of these patients, which leads to rapid re-admission to acute care facilities. In our geographical catchment area, not only have our patients been unable to be admitted to long-term care facilities, but the lower number of total beds for civil patients has also resulted in increased acuity at our hospital.

**Figure 2:**
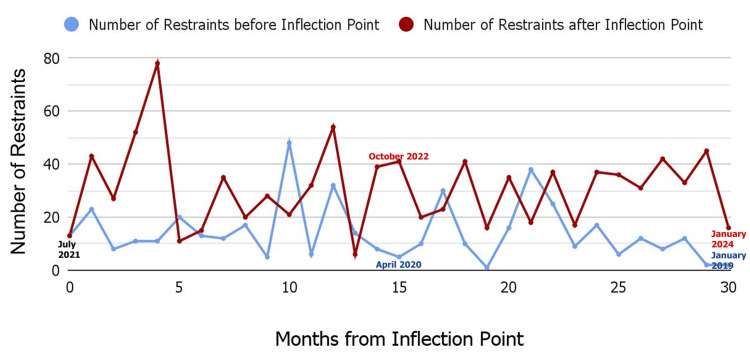
Increased number of restraints after reduction in public funding Number of restraints over 30 months before and after state hospital reducing the capacity for civil patients. Figure created by authors on Google Sheets (Google Drive, California, US). Data for the figure obtained from a quality improvement project done at Mary Washington Healthcare, Fredericksburg, US.

In our institution, we examined the date on which our local state hospital was closed to patients due to the COVID-19 pandemic. We compared the number of restraints 30 months prior to and 30 months following that date (Figure 2).

The t-value for the comparison between the two groups was 5.53, indicating a highly significant difference between the groups (means of 14.6 (SD=117.2) vs. 33.5 (SD = 235.6), p<0.001). This strongly supports the hypothesis that the difference in means between the two groups is unlikely to have occurred by chance. The large t-value, which reflects the size effect, suggests that the closure of the hospital had a significant and meaningful impact on the patients. In the three cases, all three patients would have otherwise been accepted to a state hospital had these hospitals had sufficient capacity. The lack of long-term stabilization, both legally and practically, has created a vicious cycle of recidivism, with all three patients presenting to the hospital in rapid succession. This outcome is neither in the best interest of the patients, nor of the community, family, or our local healthcare system, which faces cascading challenges. Firstly, such patients often disrupt non-psychiatric emergency room environments. Secondly, these patients require one-on-one observation, which is a strain both from a personnel perspective and financially. Lastly, the patients are stabilized to their pre-admission baseline, whereas one could argue that had the same patients stayed in the hospital for several months, their baseline presentation could have improved, reducing the need for rapid readmission. All patients had periods of significant stability. It is our impression that the pendulum of deinstitutionalization has overcorrected dramatically, leading to severe adverse outcomes for our patients.

This trend reflects the realities observed by the authors in their day-to-day care, as well as being reported by local newspapers. The issue has been identified by the community as the number one challenge facing its members. For example, The Free Lance-Star reported that 90% of patients admitted to two state hospitals are involuntary, with the lack of available long-term beds forcing the courts to send more patients to state hospitals under the forensic domain [15]. As was noted in the article, one state hospital admitted 530 civil patients in 2020, while only 42 civil patients were admitted in 2023. In another state hospital, only 30 out of 302 beds (10%) are available for civil patients. Even in geriatric psychiatric units, 60% of patients are admitted for forensic reasons. In our catchment area, 69% of the 246 patients are forensic patients [16].

The shortage of state-funded inpatient beds for individuals living with SMI has strained public relations with these hospitals. One can imagine the pressure these few state hospitals face in responding to communities frustrated by the lack of resources. The shortage of state-funded psychiatric inpatient facilities invariably creates challenges for local community hospitals. Not only do these hospitals continue to serve the patients they were traditionally obligated to care for, but they are also admitting patients who would otherwise be placed in state-funded facilities. This situation creates a host of challenges, including increased acuity in acute care inpatient hospitals.

This trend has significant unintended consequences for both hospitals and patients. First, patients with SMI who need long-term care are unable to receive the treatment they require. Second, the increased acuity in acute care psychiatric hospitals places additional strain on hospital staffing. Finally, the most unfortunate outcome is that higher functioning psychiatric patients who do not meet the criteria for involuntary treatment often leave against medical advice before they are fully stabilized, driven by anxiety stemming from the heightened acuity in these facilities.

Local hospitals can implement strategies to mitigate the impact of assuming responsibilities traditionally managed by state hospitals. For instance, at our institution, we prioritize grouping patients with similar needs within the same unit. Given the relatively large size of our facility, this approach helps reduce the likelihood of higher-functioning patients leaving without full stabilization. However, it does not alleviate staff fatigue, the increased use of restraints, or the adverse outcomes for individuals with SMI and high recidivism risk. Smaller hospitals with more limited resources may face even greater challenges, making it unlikely that local responses will fully address the issue. Consequently, the onus of finding a sustainable solution falls upon the state, which must take responsibility for the care of society’s most vulnerable members.

## Conclusions

The profound reduction in long-term psychiatric beds following deinstitutionalization has had enduring consequences for individuals, healthcare systems, and society. This decline has not coincided with a reduction in the prevalence of SMI requiring sustained treatment. As a result, many individuals who would benefit from extended hospitalization are instead redirected to the criminal justice system or left untreated and homeless. Addressing these systemic gaps will require coordinated, population-specific, and nationwide strategies. Rather than reverting to large-scale institutionalization, solutions should emphasize targeted, evidence-informed programs tailored to the complex needs of diverse patient populations through collaboration among private institutions, state hospitals, and federal systems.

Expanding psychiatric capacity within both general and state hospitals is a crucial step toward mitigating these challenges. Increasing inpatient bed availability, strengthening substance use disorder treatment networks, and developing crisis stabilization units can promote access to timely and appropriate care while reducing reliance on restrictive or emergency-based interventions. Revitalizing community-based models-such as partial hospitalization, intensive outpatient programs, and assertive community treatment (ACT) teams-would further enhance continuity of care. These measures not only improve outcomes for patients with chronic and treatment-resistant illness but also reduce staff burnout, workplace assaults, and the substantial financial strain on community hospitals. Critically, the lack of long-term stabilization options has contributed to high rates of recidivism and increased incidents of aggression toward staff as acutely ill patients cycle through short-term admissions. Sustainable reform must therefore prioritize a continuum of care that integrates acute and long-term treatment systems, ensuring safety, dignity, and recovery for both patients and providers.
